# Effects of flywheel resistance training on jump, sprint, and change-of-direction performance in basketball players: a systematic review and meta-analysis

**DOI:** 10.3389/fphys.2026.1989504

**Published:** 2026-10-06

**Authors:** Wenjing Ding, Bohui Li, Xuejun Ma

**Affiliations:** School of Education, Beijing Sport University, Beijing, China

**Keywords:** basketball, change-of-direction performance, flywheel resistance training, jump performance, sprint performance

## Abstract

**Background:**

Flywheel resistance training (FRT) has received increasing attention in athletic conditioning. Although previous studies have suggested that FRT may improve athletic performance, existing evidence has mainly been derived from mixed populations across different sports, and its sport-specific effects in basketball players remain unclear. Therefore, this study aimed to compare the effects of FRT and traditional resistance training on jump, linear sprint, and change-of-direction (COD) performance in basketball players using meta-analysis.

**Methods:**

A systematic literature search was conducted in Web of Science, PubMed, Scopus, and SPORTDiscus from database inception to August 2026. Randomized controlled trials comparing FRT with active non-flywheel resistance training and reporting jump, linear sprint, or COD performance outcomes in basketball players were included. Effect sizes were calculated as Hedges’ g with 95% confidence intervals (CI), and random-effects models were applied for meta-analysis. Between-study heterogeneity was assessed using the I^2^ statistic.

**Results:**

Seven studies involving 119 basketball players were included. Compared with traditional resistance training, FRT demonstrated a moderate beneficial effect on jump performance (Hedges’ g = 0.65, 95% CI [0.23, 1.07], p = 0.008), with no substantial heterogeneity (I^2^ = 0.0%). FRT also produced a significant beneficial effect on COD performance (Hedges’ g = −0.57, 95% CI [−1.07, −0.08], p = 0.033; I^2^ = 0.0%). For linear sprint performance, although the pooled effect favored FRT (Hedges’ g = −0.57, 95% CI [−1.20, 0.05]), the effect did not reach statistical significance (p = 0.065).

**Conclusions:**

The available evidence suggests a potential additional benefit of flywheel resistance training for jump and COD performance in basketball players, whereas evidence for an additional benefit on linear sprint performance remains uncertain. Coaches and strength and conditioning practitioners may consider FRT as a complementary strategy to traditional resistance training to enhance selected basketball-specific physical performance outcomes, particularly jump and COD performance.

## Introduction

Basketball is a high-intensity, intermittent, and multidirectional team sport in which players are required to repeatedly perform demanding actions such as acceleration, deceleration, jumping, sprinting, and rapid changes of direction during competition (Stojanović et al., 2018; Petway et al., 2020). These sport-specific demands make jumping, linear sprinting, and change-of-direction (COD) ability important indicators of physical performance in basketball players, and they are widely used to assess physical fitness and monitor training adaptations (Morrison et al., 2022). Therefore, effectively improving these three key performance qualities remains an important goal in the physical preparation of basketball players.

Among the various training methods used to improve these performance qualities, resistance training has long been regarded as an important strategy for developing strength and explosive capabilities in athletes. Previous research has shown that improvements in muscular strength and power provide an important foundation for high-intensity actions such as jumping, sprinting, and changing direction (Cormie et al., 2011; Suchomel et al., 2016). However, the adaptations induced by resistance training depend not only on the magnitude of the applied load but also on how that load is imposed. Different loading modes can alter force production, movement velocity, and the mechanical demands experienced during the concentric and eccentric phases of an exercise, thereby potentially eliciting different training adaptations (Suchomel et al., 2019). In this context, increasing attention has been directed toward training methods that modify conventional loading characteristics, among which flywheel resistance training (FRT) has attracted particular interest because of its unique inertial loading properties.

Unlike traditional resistance training, which typically relies on external mass and gravitational resistance, FRT generates resistance through the moment of inertia of a rotating flywheel. During the concentric phase, the athlete accelerates the flywheel and stores kinetic energy, which must subsequently be absorbed by actively decelerating the flywheel during the eccentric phase (Beato and Dello Iacono, 2020; Beato et al., 2024). Accordingly, the resistance encountered during FRT is not determined solely by a preselected external load but varies in accordance with the athlete’s force and velocity output. This allows force production and eccentric braking to occur continuously throughout the movement and provides the potential for substantial mechanical loading during the eccentric phase (Norrbrand et al., 2010; Martinez-Aranda and Fernandez-Gonzalo, 2017). However, FRT should not be considered synonymous with eccentric overload, as the occurrence and magnitude of eccentric overload depend on factors such as flywheel inertia, movement execution, braking strategy, and the athlete’s ability to accelerate and decelerate the flywheel. Because of these loading characteristics, FRT may not only contribute to the development of basic strength but may also influence the ability to rapidly produce, absorb, and reapply force across successive concentric and eccentric actions.

These characteristics are theoretically relevant to several key movement demands in basketball. Jumping requires athletes to rapidly transition from braking to upward propulsion; short-distance sprinting requires high levels of propulsive force to be generated within limited ground-contact times; and COD actions require athletes to reduce their existing velocity and control whole-body momentum before rapidly reaccelerating in a new direction (Nagahara et al., 2018; Harper et al., 2022; Kozinc, 2022). These tasks therefore depend not only on the ability to generate force rapidly but also on the effective absorption and subsequent reapplication of force. The continuous concentric–eccentric loading and active braking demands imposed by FRT may therefore correspond well with the mechanical requirements of these movement tasks. Previous systematic reviews and meta-analyses have shown that chronic FRT can improve muscular strength, power, and several aspects of athletic performance (Maroto-Izquierdo et al., 2017; Petré et al., 2018; de Keijzer et al., 2022). Consequently, for basketball players, the more practically relevant question is not simply whether FRT can improve general strength, but whether these adaptations translate into meaningful improvements in jumping, sprinting, and COD performance.

A growing body of research has begun to examine the long-term effects of FRT on athletic performance. A systematic review and meta-analysis by Raya-González et al. (2021) reported that FRT improved jumping, sprinting, and COD performance, providing initial quantitative evidence to support its use in athletic preparation. Other systematic reviews and umbrella reviews have similarly supported the potential of FRT to enhance strength, power, and selected performance outcomes (Maroto-Izquierdo et al., 2017; de Keijzer et al., 2022). However, the existing evidence has largely been derived from mixed samples including athletes from soccer, handball, basketball, and other sports, with some studies also involving healthy or physically active participants. This is an important limitation because the transfer of resistance-training adaptations to athletic performance is influenced by the mechanical and task-specific demands of the target sport. Basketball is characterized by frequent jumping, rapid acceleration and deceleration, and repeated changes of direction, all of which require athletes to rapidly generate, absorb, and reapply force. These demands may interact differently with the concentric–eccentric loading and active braking characteristics of FRT than in sports with different locomotor and mechanical requirements. Therefore, pooled effects derived from heterogeneous sporting populations may not fully represent the performance adaptations induced by FRT in basketball players.

To address this issue, Younes-Egana et al. (2023) specifically focused on basketball players and systematically reviewed the effects of FRT on their physical performance. Overall, the available evidence suggested that FRT may improve key outcomes such as jumping, sprinting, and COD performance, providing more sport-specific support for its application in basketball conditioning. However, that review primarily synthesized the available findings qualitatively and did not use meta-analytic methods to estimate pooled effect sizes or the associated statistical uncertainty for jumping, sprinting, and COD outcomes separately. Given the generally small sample sizes of individual studies and the inconsistency of some findings, relying solely on whether individual studies reach statistical significance makes it difficult to determine the true overall effect of FRT on these performance outcomes. Therefore, a quantitative synthesis of the available basketball-specific evidence is warranted to determine whether the performance benefits reported across broader athletic populations are also evident in basketball players and to quantify the magnitude and uncertainty of these effects across jumping, sprinting, and COD performance.

Accordingly, the purpose of this systematic review and meta-analysis was to quantitatively compare the effects of flywheel resistance training with traditional resistance training on jump, linear sprint, and COD performance in basketball players. By synthesizing basketball-specific evidence, this study aimed to determine whether FRT provides additional performance benefits beyond traditional resistance training and to quantify the magnitude and uncertainty of these effects across the three outcomes.

## Methods

### Protocol and registration

This systematic review and meta-analysis was conducted and reported in accordance with the PRISMA 2020 statement and aimed to evaluate the effects of flywheel resistance training on jump, sprint, and COD performance in basketball players (Page et al., 2021). The review protocol was prospectively registered with the International Prospective Register of Systematic Reviews (PROSPERO; registration number: CRD420261491727).

### Search strategy and selection process

Web of Science Core Collection, PubMed, Scopus, and SPORTDiscus were systematically searched to identify studies evaluating the effects of flywheel resistance training on jump, sprint, and COD performance in basketball players. The searches covered the period from database inception to 28 August 2026. The search strategy was developed around two core concepts, namely “flywheel resistance training” and “basketball players,” with keywords and Boolean operators adapted as appropriate for each database. The main search terms related to flywheel resistance training included flywheel training, flywheel resistance training, flywheel eccentric training, isoinertial training, and eccentric overload, whereas basketball-related terms included basketball, basketball player, and basketball athlete. The complete search strategies for each database are provided in the **Supplementary Materials**. In addition, the reference lists of the included studies and relevant systematic reviews were manually screened to identify potentially eligible studies that may have been missed by the database searches.

All retrieved records were imported into EndNote 20 (Clarivate, Philadelphia, PA, USA), and duplicate records were removed. Two reviewers (W.J.D and B.H.L) independently screened the titles and abstracts according to the predefined eligibility criteria and subsequently assessed the full texts of potentially eligible studies. Any disagreements between the two reviewers were resolved through discussion; if consensus could not be reached, a third reviewer (X.J.M) was consulted for adjudication.

### Eligibility criteria

Eligibility criteria were established *a priori* according to the PICOS framework. Studies were considered eligible if they met the following criteria: participants were basketball players, regardless of sex, age, or competitive level; the intervention consisted of a longitudinal flywheel resistance training program using flywheel-based inertial resistance; the study included at least one active non-flywheel comparator, such as traditional resistance training, barbell-based resistance training, another resistance-training intervention that did not involve flywheel-based inertial resistance; at least one outcome related to jump, sprint, or COD performance was reported; and sufficient quantitative data were available to calculate effect sizes and their sampling variances. Only original, peer-reviewed, full-text longitudinal controlled intervention studies were included.

Studies were excluded if they examined only the acute effects of a single bout of flywheel exercise rather than adaptations following a training period; did not include an active non-flywheel training comparator, including studies in which the control group only maintained regular basketball training or received no additional resistance-training intervention; compared only different flywheel loads, exercise modes, or training prescriptions across intervention groups; included participants from multiple sports without separately extractable data for basketball players; did not report outcomes related to jump, sprint, or COD performance; provided insufficient data to calculate effect sizes and their sampling variances; or were reviews, conference abstracts, theses, case reports, editorials, or other non-original or non-peer-reviewed publications.

### Risk of bias and certainty of evidence

The risk of bias of the included studies was assessed using the Cochrane Risk of Bias 2 (RoB 2) tool (Sterne et al., 2019). RoB 2 evaluates potential bias across five domains: (1) bias arising from the randomization process; (2) bias due to deviations from intended interventions; (3) bias due to missing outcome data; (4) bias in measurement of the outcome; and (5) bias in selection of the reported result. Based on the signaling questions and the corresponding RoB 2 algorithms, each domain was judged as “low risk of bias,” “some concerns,” or “high risk of bias,” and an overall risk-of-bias judgement was subsequently assigned to each study.

Risk-of-bias assessments were independently conducted by two reviewers (W.J.D and B.H.L). Any disagreements were initially resolved through discussion; when consensus could not be reached, a third reviewer (X.J.M) was consulted for adjudication. The final risk-of-bias assessments were presented graphically. The certainty of evidence for each outcome was further evaluated using the Grading of Recommendations Assessment, Development and Evaluation (GRADE) approach, considering risk of bias, inconsistency, indirectness, imprecision, and publication bias. The certainty of evidence was classified as high, moderate, low, or very low (Guyatt et al., 2011).

### Data extraction

Two reviewers (W.J.D and B.H.L) independently extracted data using a predesigned data extraction form. The extracted information included study characteristics (first author, year of publication, and study design), participant characteristics (sample size, sex, age, and competitive level), intervention characteristics (exercise modality, intervention duration, weekly training frequency, training volume, and flywheel inertia or other loading parameters), comparator training protocols, and outcome data related to jump, sprint, and COD performance. Any disagreements between the two reviewers were resolved through discussion; if consensus could not be reached, a third reviewer (X.J.M) was consulted for adjudication. For continuous outcomes, group sample sizes, means, and standard deviations were extracted for subsequent effect-size calculation. When relevant data were not reported directly, values were extracted or derived from the main text, tables, **Supplementary Materials**, or other available information. When essential data required for effect-size calculation could not be obtained, the corresponding authors were contacted; studies for which the required data remained unavailable after follow-up were excluded from the quantitative synthesis. Numerical data reported only in graphical form were digitized using WebPlotDigitizer or GetData Graph Digitizer (version 2.26).

### Statistical analysis

All statistical analyses were performed using R version 4.4.2 (R Foundation for Statistical Computing, Vienna, Austria) with the metafor package (Viechtbauer, 2010). Separate meta-analyses were conducted for jump, sprint, and COD performance. Intervention effects were calculated from the pre-to-post changes in the intervention and comparator groups and expressed as standardized mean differences corrected for small-sample bias, using Hedges’ g with 95% confidence intervals (CIs) (Morris, 2008). Study-reported standard deviations of change scores were used when available. When these were not reported, they were estimated from the pre- and post-intervention standard deviations. As study-specific pre–post correlation coefficients were not available for the outcomes requiring estimation, a correlation of r = 0.50 was assumed in the primary analysis (Higgins et al., 2024). To examine the influence of this assumption, sensitivity analyses were conducted using alternative pre–post correlations of r = 0.30 and r = 0.70. When a study reported multiple eligible measures within the same outcome domain, all corresponding effect sizes were retained. Because effect sizes derived from the same study were not assumed to be statistically independent, a three-level random-effects model was initially fitted to account for sampling variance (Level 1), within-study variance between effect sizes (Level 2), and between-study variance (Level 3) (Assink and Wibbelink, 2016). Likelihood-ratio tests were used to compare the full three-level model with the corresponding reduced two-level models. When the three-level model provided a significantly better fit, it was retained for the primary analysis; otherwise, a conventional two-level random-effects model was used to pool effect sizes. Variance components were estimated using restricted maximum likelihood (REML), with statistical inference based on the t distribution. In addition to the pooled effect estimates and 95% CIs, 95% prediction intervals (PIs) were calculated to describe the range in which the true effect of a future comparable study might be expected to lie (IntHout et al., 2016). Statistical heterogeneity was assessed using Cochran’s Q test and the I^2^ statistic, with values of approximately 25%, 50%, and 75% interpreted as low, moderate, and high heterogeneity, respectively (Higgins et al., 2003). Because fewer than 10 studies were included for each outcome, funnel plots and statistical tests for funnel-plot asymmetry were not performed (Sterne et al., 2011). All tests were two-sided, with statistical significance set at p < 0.05.

## Results

### Study selection

A total of 94 records were identified through the database searches, including 16 from PubMed, 23 from Web of Science, 28 from Scopus, and 27 from SPORTDiscus. After the removal of 46 duplicate records, 48 unique records remained for title and abstract screening. Of these, 37 records were excluded because they did not meet the predefined eligibility criteria, leaving 11 reports for full-text retrieval. All 11 full-text reports were successfully retrieved and assessed for eligibility. Following full-text screening, five reports were excluded: two investigated the acute effects of flywheel resistance exercise, two compared different flywheel training protocols without an eligible non-flywheel comparator, and one included a mixed participant population from which basketball-specific data could not be extracted. Consequently, six studies identified through the database searches met the eligibility criteria. In addition, one eligible study was identified through manual screening of the reference lists of the included studies and relevant systematic reviews and was subsequently retrieved and assessed for eligibility. Ultimately, a total of seven studies were included in the systematic review and meta-analysis. The detailed study selection process is presented in **Figure 1**.

**Figure 1:**
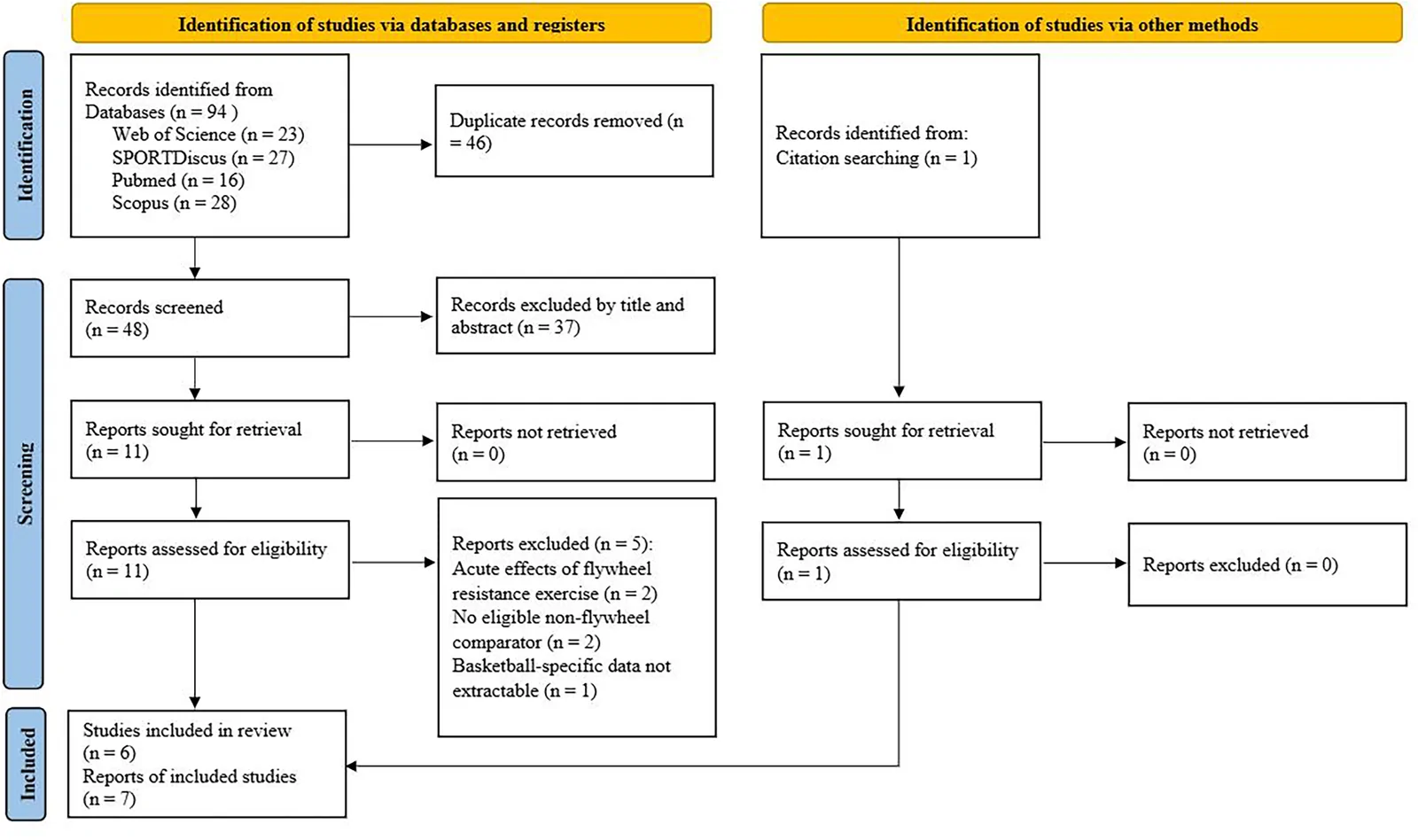
Preferred reporting items for systematic reviews and meta-analyses (PRISMA) 2020 flow diagram of the study selection process.

### Study characteristics

A total of 119 basketball players were included across the seven studies, comprising collegiate, youth, amateur, and professional athletes. Four studies included female players (O’Brien et al., 2020; Xie et al., 2024; Peng et al., 2025; Xue et al., 2025), two included male players (Stojanović et al., 2021; Shimizu et al., 2025), and one included both male and female players (Cabanillas et al., 2020). Flywheel resistance training interventions lasted 4–8 weeks and were performed 1–2 times per week. Training programs were predominantly based on lower-body multi-joint exercises, with the squat or half squat being the most commonly used movements; some studies also incorporated Romanian deadlifts and single-leg squats (Stojanović et al., 2021; Xue et al., 2025). Considerable variation was observed in flywheel loading. Five studies used a fixed inertia of 0.075 kg·m^2^ (O’Brien et al., 2020; Stojanović et al., 2021; Xie et al., 2024; Peng et al., 2025; Shimizu et al., 2025), one used 0.0332 kg·m^2^ (Cabanillas et al., 2020), and one prescribed inertia individually within a range of approximately 0.010–0.050 kg·m^2^ according to individual loading characteristics (Xue et al., 2025). Training volume generally consisted of 8–10 repetitions per set, with some studies progressively increasing the number of sets over the intervention period (Cabanillas et al., 2020; Stojanović et al., 2021). Comparator conditions primarily involved traditional free-weight or barbell resistance training, with external loads prescribed relative to one-repetition maximum (1RM) in several studies. Outcome measures covered jump, linear sprint, and COD performance. Jump performance was assessed in six studies (Cabanillas et al., 2020; O’Brien et al., 2020; Stojanović et al., 2021; Xie et al., 2024; Shimizu et al., 2025; Xue et al., 2025), sprint performance in five studies (Cabanillas et al., 2020; O’Brien et al., 2020; Stojanović et al., 2021; Peng et al., 2025; Xue et al., 2025), and COD performance in four studies (O’Brien et al., 2020; Stojanović et al., 2021; Peng et al., 2025; Xue et al., 2025). Countermovement jump and squat jump were the most commonly used jump tests, sprint distances ranged from 5 to 30 m, and COD performance was mainly assessed using the 505 test, T-test, and Lane Agility Test. The characteristics of the included studies are presented in **Table 1**.

**Table 1:** Characteristics of the included studies.

Study	Population characteristics				Intervention characteristics						Outcome measures
	Sample size	Sex	Age, y	Competitive level	Duration	Frequency	Flywheel exercise	Inertia/load	Sets × reps	Comparator	
(Peng et al., 2025)	FET: 9; BST: 10	Female	FET: 20.7 ± 2.0; BST: 20.2 ± 2.6	Collegiate	6 week	2 sessions/wk	Squat	0.075 kg·m^2^	4 × (2 + 8) (2 spin-up + 8 working reps)	Barbell squat (80% 1RM; 4 × 8)	20-m sprint; 505 agility test; Lane agility test
(Xue et al., 2025)	Flywheel: 8; Barbell: 8	Female	Flywheel: 21.00 ± 1.69; Barbell: 21.13 ± 1.89	Collegiate	8 week	2 sessions/wk	Back squat; hard pull; single-leg squat	Individualized from 0.010, 0.025, or 0.050 kg·m^2^ (velocity-matched to 80% 1RM)	NR	Traditional barbell back squat; hard pull; single-leg squat (80% 1RM)	CMJ; 30-m sprint; T-test; 17 × 15-m shuttle run
(Shimizu et al., 2025)	FWT: 11; CON: 9	Male	FWT: 19.5 ± 1.2; CON: 20.1 ± 1.3	Collegiate	8 week	2 sessions/wk	Squat	0.075 kg·m^2^	5 × 8	Regular basketball training + traditional resistance training (1 session/wk); no flywheel training	SJ; CMJ; 20-cm drop jump; 40-cm drop jump; RSI (20-cm and 40-cm drop jumps)
(Xie et al., 2024)	FRST: 9; TRST: 10	Female	FRST: 20.7 ± 2.0; TRST: 20.2 ± 2.6	Collegiate	6 week	2 sessions/wk	Squat	0.075 kg·m^2^	4 × (2 + 8) (2 spin-up + 8 working reps)	Barbell squat (80% 1RM; 4 × 8)	CMJ; RSI (45-cm drop jump); Running Vertical Jump; Eccentric Utilization Ratio (CMJ/SJ)
(Stojanović et al., 2021)	FST: 12; TST: 12	Male	FST: 17.58 ± 0.52; TST: 17.52 ± 0.58	Junior (regional level)	8 week	1–2 sessions/wk	Half squat; Romanian deadlift	0.075 kg·m^2^	wk 1–2: 2 × 8; wk 3–6: 3 × 8; wk 7–8: 4 × 8 per exercise (+2 uncounted spin-up reps/set)	TST: Free-weight half squat + Romanian deadlift	CMJ; 5-m sprint split; 20-m sprint; agility T-test
(O’Brien et al., 2020)	FIT: 11; TET: 9	Female	FIT: 23.17 ± 5.55; TET: 24.18 ± 6.56	Amateur	4 week	2 sessions/wk	NR	0.075 kg·m^2^	4 × 10 (first 2 reps/set momentum-building; 8 working reps)	Tempo-eccentric barbell back squat (65% 1RM; 4 × 8; 2-s concentric/4-s eccentric)	CMJ; SJ; 10-m sprint; 505 test
(Cabanillas et al., 2020)	Inertial: 4; Traditional: 4	Mixed	21.3 ± 3.45	Professional	6 week	1 sessions/wk	Half squat	0.0332 kg·m^2^	wk 1–2: 4 × 10; wk 3–4: 5 × 10; wk 5–6: 6 × 10	Traditional free-weight half squat (10 reps of 14 possible at maximal speed)	CMJ; 30-m sprint

1RM, one-repetition maximum; BST, barbell squat training; CMJ, countermovement jump; CON, control; FET, flywheel eccentric training; FIT, flywheel inertial training; FRST, flywheel resistance squat training; FST, flywheel strength training; FWT, flywheel training; NR, not reported; PPO, peak power output; RSI, reactive strength index; SJ, squat jump; TET, tempo eccentric training; TRST, traditional resistance squat training; TST, traditional strength training; wk, week(s).

### Study quality assessment

The RoB 2 assessment is presented in **Figure 2**. All seven included studies were judged as having some concerns overall (Cabanillas et al., 2020; O’Brien et al., 2020; Stojanović et al., 2021; Xie et al., 2024; Peng et al., 2025; Shimizu et al., 2025; Xue et al., 2025). The main sources of potential bias were the randomization process and selection of the reported result. Five studies raised some concerns regarding the randomization process (Cabanillas et al., 2020; O’Brien et al., 2020; Stojanović et al., 2021; Shimizu et al., 2025; Xue et al., 2025), whereas Peng et al. (2025) and Xie et al. (2024) were rated as low risk in this domain. All seven studies raised some concerns regarding selection of the reported result, primarily because the available prospective information was insufficient to verify that the reported outcome measurements and analyses had been fully prespecified before the results were known. All studies were rated as low risk for deviations from intended interventions and measurement of outcomes. For missing outcome data, five studies were rated as low risk (Cabanillas et al., 2020; O’Brien et al., 2020; Stojanović et al., 2021; Shimizu et al., 2025; Xue et al., 2025), whereas two studies raised some concerns (Xie et al., 2024; Peng et al., 2025).

**Figure 2:**
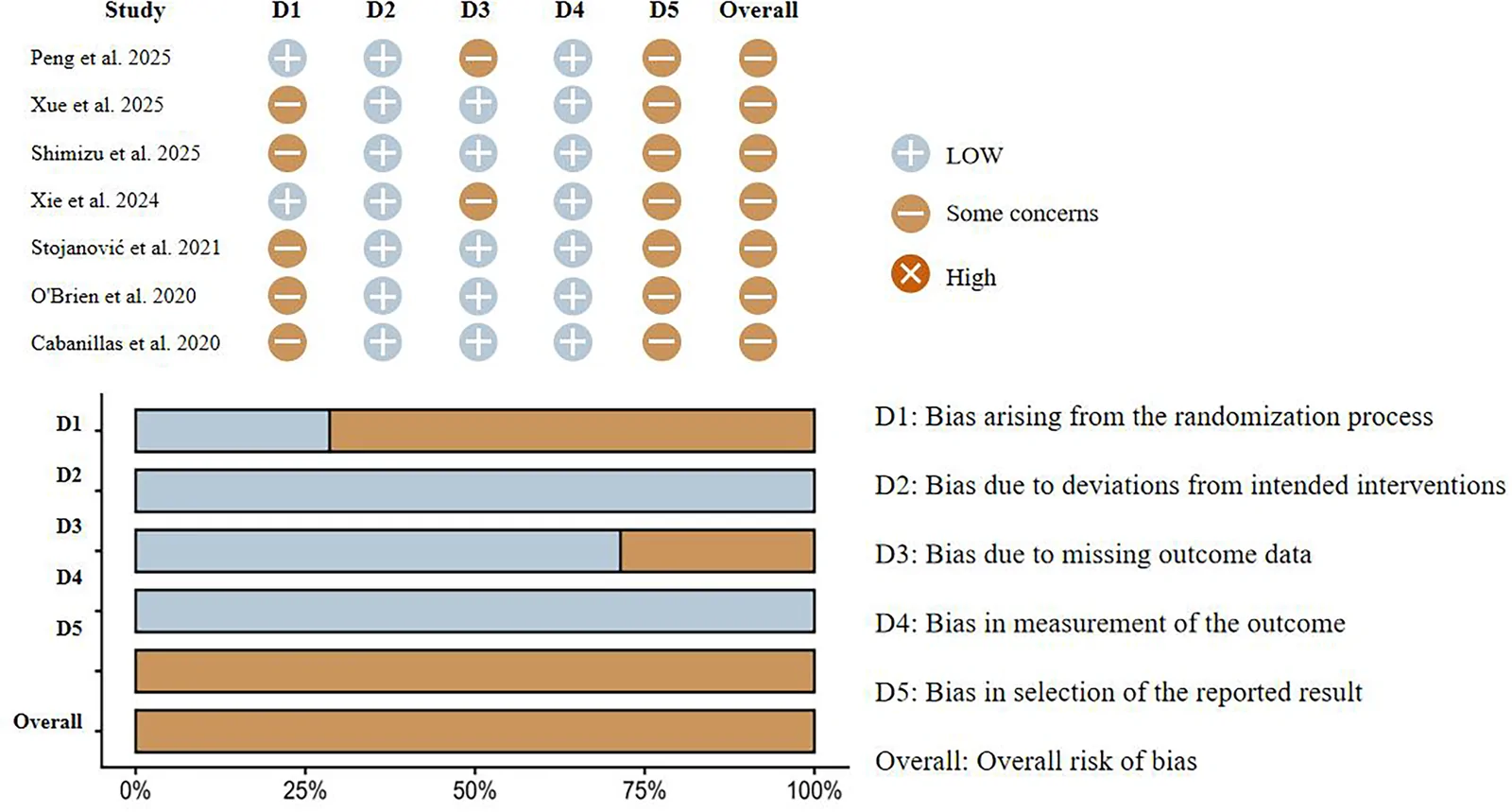
Risk-of-bias assessment of the included studies.

### Main effect

For interpretation, positive Hedges’ g values indicated effects favoring FRT for jump performance, whereas negative values favored FRT for sprint and COD performance, where lower completion times reflect better performance. The meta-analyses comprised eight effect sizes from six studies for jump, six effect sizes from five studies for sprint, and five effect sizes from four studies for COD. Because several studies contributed multiple effect sizes within the same outcome domain, a three-level random-effects model was initially fitted to account for potential dependence among effects originating from the same study. However, both the within-study and between-study variance components were estimated as zero. Constraining the within-study variance component to zero did not result in a significant deterioration in model fit (p > 0.05), while the reduced model yielded lower AIC and AICc values. Therefore, based on model parsimony, the two-level random-effects model was used for the subsequent main-effect analyses. Detailed model comparisons are presented in **Supplementary Table 1**.

### Effects of flywheel resistance training on jump performance

Flywheel resistance training showed a moderate favorable effect on jump performance (Hedges’ g = 0.65, 95% CI [0.23, 1.07], p = 0.008), with negligible heterogeneity (I^2^ = 0.0%, τ^2^ < 0.001; Q(7) = 7.48, p = 0.380). The 95% prediction interval ranged from 0.23 to 1.07, and the certainty of evidence was rated as moderate according to GRADE (**Figure 3**). Sensitivity analyses using alternative pre–post correlations of r = 0.30 and r = 0.70 yielded the same statistical conclusion, with the pooled effect remaining significant under both assumptions (**Supplementary Table 2**).

**Figure 3:**
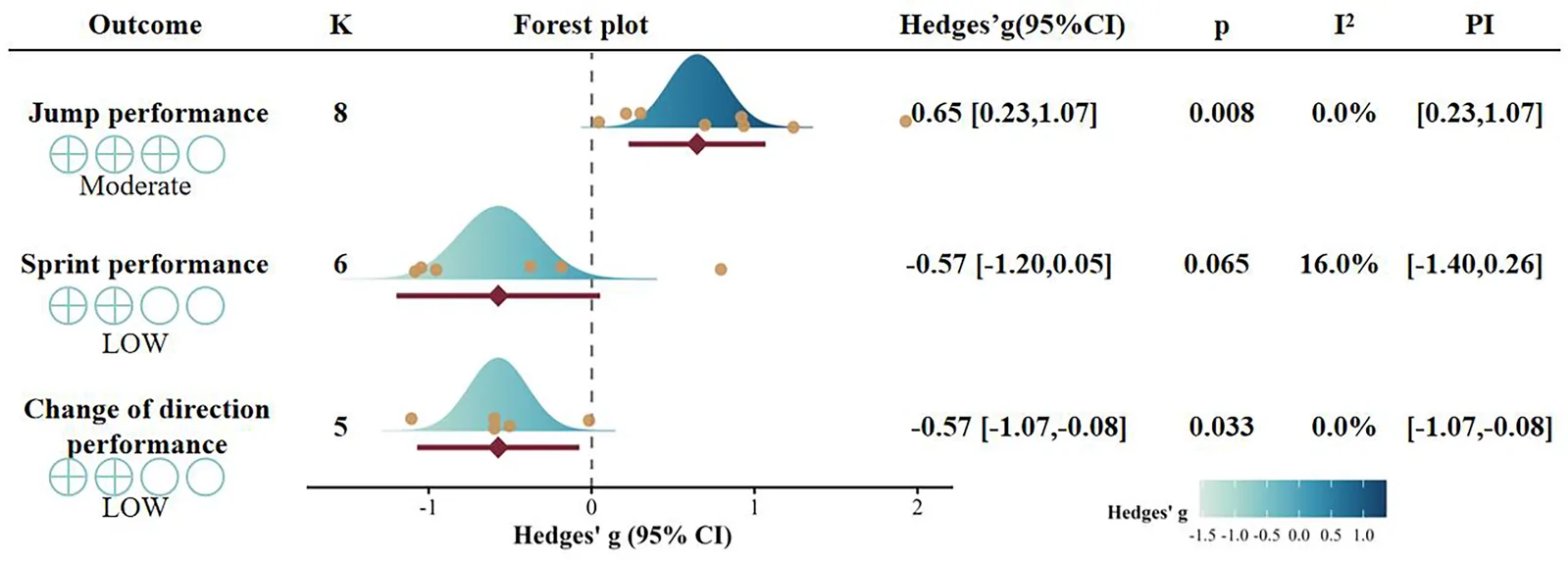
Summary of the effects of flywheel resistance training on jump, sprint, and change-of-direction performance. K, number of effect sizes; CI, confidence interval; PI, prediction interval; I^2^, percentage of total variability attributable to heterogeneity. Orange dots represent individual effect sizes, burgundy diamonds represent pooled effect estimates, and horizontal burgundy lines represent the corresponding 95% CIs. The dashed vertical line indicates the null effect (Hedges’ g = 0). Certainty of evidence was assessed using GRADE: ⊕⊕⊕○, moderate certainty; ⊕⊕○○, low certainty.

### Effects of flywheel resistance training on sprint performance

For sprint performance, flywheel resistance training showed a moderate favorable effect; however, the 95% confidence interval crossed zero and the pooled effect did not reach statistical significance (Hedges’ g = −0.57, 95% CI [−1.20, 0.05], p = 0.065). Between-study heterogeneity was low (I^2^ = 16.0%, τ^2^ = 0.045; Q(5) = 7.21, p = 0.205), and the 95% prediction interval ranged from −1.40 to 0.26. The certainty of evidence was rated as low according to GRADE (**Figure 3**). The pooled effect remained non-significant in sensitivity analyses using r = 0.30 and r = 0.70, supporting the uncertainty of the primary estimate (**Supplementary Table 2**).

### Effects of flywheel resistance training on change-of-direction performance

For COD performance, flywheel resistance training showed a moderate favorable effect, with the pooled estimate reaching statistical significance (Hedges’ g = −0.57, 95% CI [−1.07, −0.08], p = 0.033). Between-study heterogeneity was negligible (I^2^ = 0.0%, τ^2^ = 0; Q(4) = 2.98, p = 0.562), and the 95% prediction interval ranged from −1.07 to −0.08. The certainty of evidence was rated as low according to GRADE because of concerns regarding risk of bias and imprecision associated with the limited evidence base. (**Figure 3**). Sensitivity analyses using r = 0.30 and r = 0.70 produced the same statistical conclusion, with the pooled effect remaining significant under both assumptions (**Supplementary Table 2**).

## Discussion

To our knowledge, this is the first meta-analysis to quantitatively synthesize the effects of flywheel resistance training specifically in basketball players. Compared with traditional resistance training, flywheel resistance training produced moderate favorable effects on jump and COD performance, whereas a similarly favorable effect on sprint performance did not reach statistical significance. Overall, these findings support the potential use of flywheel resistance training to improve jump and COD performance in basketball players; however, its effect on sprint performance remains uncertain.

These findings partly align with the earlier basketball-specific systematic review by Younes-Egana et al. (2023), which qualitatively suggested beneficial effects of FRT on jumping, sprinting, and COD performance. The present meta-analysis extends that review by quantitatively estimating the effects for each performance domain. While the pooled findings support additional benefits of FRT for jump and COD performance compared with traditional resistance training, the additional benefit for linear sprint performance was not statistically significant and therefore remains uncertain.

Among the three performance outcomes, jump performance showed the clearest favorable pooled effect of flywheel resistance training. The present meta-analysis identified a moderate favorable effect on jump performance in basketball players, accompanied by low between-study heterogeneity. However, given the small number of contributing studies, this low heterogeneity estimate should be interpreted cautiously because the statistical power to detect between-study heterogeneity was limited. This finding is consistent with previous evidence. Raya-González et al. (2021) reported an almost identical effect magnitude for jump performance in a meta-analysis across different sports, and a subsequent systematic review and meta-analysis focusing specifically on jump outcomes further supported the beneficial effects of flywheel resistance training on measures such as countermovement jump performance (Shimizu et al., 2024). The consistency of these findings across studies and populations suggests that the improvement in jump performance may reflect a combination of neural, muscular, and task-specific adaptations rather than a single physiological mechanism. Contemporary models of resistance-training adaptation emphasize interacting changes across supraspinal, spinal/motoneuronal, and muscular levels, while the extent to which specific training variables translate into particular neuromechanical adaptations remains incompletely understood (Lecce et al., 2026).

During flywheel exercise, athletes are required to actively accelerate the rotating system during the concentric phase and subsequently decelerate it during the eccentric phase, thereby imposing substantial demands on concentric force production, eccentric braking, and the rapid transition between eccentric and concentric actions. Importantly, these mechanical characteristics should not be interpreted as evidence that eccentric overload necessarily occurs during all flywheel exercises, as its magnitude depends on factors such as inertia, exercise selection, and braking strategy (Beato et al., 2024). Evidence from basketball players provides further support for this interpretation. Shimizu et al. (2025) observed concurrent improvements in squat jump, drop jump, and reactive strength index following flywheel squat training, together with increases in both concentric and eccentric peak power. Given that squat jump performance relies predominantly on concentric force and power production, whereas drop jump performance and reactive strength index are more dependent on rapid stretch–shortening cycle function (Van Hooren and Zolotarjova, 2017; Ramirez-Campillo et al., 2023), the simultaneous improvement in these outcomes suggests that the effects of flywheel training on jumping may reflect coordinated adaptations in force production, force absorption, and rapid force transition rather than an isolated eccentric stimulus. However, because these mechanisms were not directly assessed in most of the included studies, this interpretation should be regarded as a potential explanation rather than a demonstrated mechanism. In addition, most interventions included in the present review employed multi-joint lower-body exercises such as squats or half squats, whereas the primary jump outcomes were countermovement and squat jumps. The similarity between these training and testing tasks in terms of hip, knee, and ankle extension patterns and vertical force production may therefore facilitate the transfer of training adaptations. Collectively, the relatively close mechanical correspondence between the training characteristics of flywheel resistance training and the demands of jumping may help explain the consistent improvements in jump performance observed in the present meta-analysis.

Compared with jump performance, the advantage of flywheel resistance training for sprint performance was less clearly supported. Although the pooled estimate favored flywheel resistance training with a moderate effect magnitude, the 95% CI crossed zero, indicating that current evidence is insufficient to establish a clear advantage over traditional resistance training for improving sprint performance in basketball players. This uncertainty is also reflected in the 95% prediction interval (−1.40 to 0.26), which spans effects ranging from a substantial benefit of FRT to a small effect favoring traditional resistance training. Importantly, this finding reflects the between-group contrast between the two training modalities rather than whether flywheel training itself can improve sprint performance. Basketball-specific studies have reported improvements in short-distance sprint performance following flywheel training, particularly over 5- and 10-m distances, although findings at longer sprint distances have been less consistent (O’Brien et al., 2020; Stojanović et al., 2021; Peng et al., 2025). Thus, the non-significant pooled effect is more appropriately interpreted as indicating that the additional benefit of flywheel training over traditional resistance training for sprint performance has not yet been consistently demonstrated, rather than as evidence of an absence of training effect. Traditional resistance training itself can enhance lower-limb strength, power, and acceleration capacity (Suchomel et al., 2016; Hughes et al., 2023); consequently, when two potentially effective training strategies are compared directly, between-group differences are likely to be smaller than those observed when flywheel training is compared with a non-training control. Consistent with this interpretation, a recent meta-analysis in team-sport athletes found no clear advantage of flywheel over traditional resistance training for 10- or 20-m sprint performance (Eidiyan-Kakhki et al., 2026).

The uncertainty surrounding this between-group advantage may also reflect the specific movement demands of basketball and the specificity of training transfer. High-intensity locomotion during basketball competition is characterized primarily by repeated short-distance accelerations, decelerations, and changes of direction, whereas longer-distance linear sprinting occurs relatively infrequently (Stojanović et al., 2018). Therefore, sprint tests conducted over different distances may represent distinct performance characteristics, with shorter sprint distances primarily reflecting acceleration capacity, while longer distances involve greater contributions from maximal velocity development (Nagahara et al., 2018). Basketball-specific evidence also suggests that sprint adaptations following flywheel training may be distance dependent. For example, Stojanović et al. (2021) reported improvements in 5-m sprint performance following flywheel training, whereas no significant improvements were observed over 20 m. The sprint distances included in the present meta-analysis ranged from 5 to 30 m, thereby encompassing both the initial acceleration phase and subsequent velocity development phase, which may have increased uncertainty in the pooled effect estimate. Moreover, most flywheel training interventions included multi-joint lower-body exercises such as squats and half squats. However, whether the resulting adaptations in strength and power can effectively transfer to linear sprint performance may depend on the athlete’s ability to express these adaptations under the specific mechanical and temporal constraints of sprinting, including the direction of force application, timing of force production, and the ability to generate high levels of force within very short ground-contact times (Young, 2006). Therefore, improvements in strength and power capacity do not necessarily translate directly into enhanced sprint performance, and the degree of mechanical correspondence between training adaptations and testing tasks may represent an important determinant of transfer effectiveness. From this perspective, the moderate favorable point estimate and the non-significant between-group effect observed in the present meta-analysis are not contradictory. Rather, these findings suggest that flywheel resistance training may improve sprint performance in basketball players, but its additional benefit compared with traditional resistance training remains dependent on basketball-specific movement demands, sprint distance, and the specificity of training transfer.

Compared with the greater uncertainty observed for sprint performance, flywheel resistance training produced a moderate favorable effect on COD performance in basketball players, with no statistical heterogeneity detected (I^2^ = 0.0%). Because only four studies contributed to this outcome, however, the absence of detected heterogeneity should be interpreted cautiously, as the statistical power to detect between-study heterogeneity was limited. Moreover, the certainty of evidence for this outcome was rated as low according to GRADE, which further limits confidence in the statistically significant pooled estimate. This finding is consistent with previous evidence across sporting populations. Chaabene et al. (2022), in a meta-analysis directly comparing flywheel with traditional resistance training, reported an additional benefit of flywheel training for COD performance, while a more recent meta-analysis in team-sport athletes reported a comparable effect magnitude (Zhang et al., 2026). Basketball-specific studies provide further support for this finding. Stojanović et al. (2021) reported greater improvements in the T-test following flywheel training than after volume-matched traditional resistance training in youth basketball players, whereas Peng et al. (2025) found that six weeks of flywheel squat training produced greater improvements in the 505 test than barbell squat training performed at 80% 1RM.

The observed between-group advantage may be related to the combined demands of force absorption and subsequent force production during COD tasks. Unlike linear sprinting, rapid directional changes require athletes to decelerate the center of mass at relatively high approach velocities, tolerate and absorb substantial braking forces, redirect force application, and subsequently reaccelerate in a new direction. Recent biomechanical evidence indicates that superior COD performance is associated not only with greater propulsive force but also with greater braking force, shorter ground-contact times, and higher entry and exit velocities (Dos’Santos et al., 2020; Harper et al., 2022). These demands show a degree of mechanical correspondence with flywheel resistance exercise. Following active acceleration of the flywheel during the concentric phase, athletes must actively decelerate the rotating system during the return phase, repeatedly coupling force production with force absorption. Although this process does not necessarily result in eccentric overload in every flywheel exercise, the repeated requirement for active eccentric braking may provide a distinct stimulus relevant to braking capacity and the subsequent reapplication of force compared with conventional gravitational resistance exercise (Beato et al., 2024).

This transfer may be particularly relevant to basketball, where high-intensity locomotor actions frequently involve abrupt stops, cuts, lateral movements, and repeated deceleration–reorientation–reacceleration sequences rather than isolated linear acceleration (Stojanović et al., 2018). Accordingly, the between-group advantage observed in the present meta-analysis may reflect not simply greater gains in lower-limb strength, but a closer mechanical correspondence between the demands imposed by flywheel resistance training and those of basketball-specific directional changes. Force absorption, braking, and reacceleration may contribute to this correspondence, although these adaptations were not directly assessed in most of the included studies and therefore remain potential rather than demonstrated explanations for the observed effect. Nevertheless, this effect should not be interpreted as evidence that flywheel resistance training improves all forms of COD performance to the same extent. Peng et al. (2025), for example, reported improvements in the 505 test but not in the Lane Agility Test within the same basketball cohort, while O’Brien et al. (2020) did not observe a clear improvement in COD performance. Recent meta-analytic evidence in team-sport athletes has likewise indicated variation in responsiveness across different COD tests (Zhang et al., 2026). Such findings suggest that training transfer may also depend on factors including COD angle, test structure, approach velocity, and task-specific technical demands (Nimphius et al., 2018; Dos’Santos et al., 2020). Therefore, the present findings are best interpreted as suggesting a potential advantage of flywheel resistance training over traditional resistance training for COD performance in basketball players, although this advantage may be task-specific rather than generalizable across all COD demands.

Overall, the present findings indicate that the effects of flywheel resistance training are not uniform across different physical performance outcomes in basketball players. Compared with traditional resistance training, flywheel resistance training produced moderate favorable effects on jump and COD performance, whereas a similarly favorable point estimate was observed for linear sprint performance but without clear evidence of an additional benefit.

Given the frequent demands for jumping, abrupt stopping, deceleration, directional changes, and reacceleration in basketball, these findings suggest that the effects of flywheel resistance training may transfer more readily to jump and COD tasks involving rapid force production, force absorption, and subsequent force reapplication. In contrast, linear sprinting in basketball typically occurs over relatively short distances and is also influenced by horizontal force production, ground-contact time, and sprint-specific technical factors. Consequently, flywheel training programs dominated by lower-body squat-based exercises may not consistently provide additional benefits over traditional resistance training for linear sprint performance. Taken together, the value of flywheel resistance training in basketball conditioning may lie more in its task-specific contribution to selected performance qualities than in serving as a comprehensive replacement for traditional resistance training.

### Limitations

This study has several limitations. First, the direct evidence on flywheel resistance training in basketball players remains limited, with a small number of included studies and generally modest sample sizes. Most participants were collegiate or youth players, whereas only one small study included professional players (n = 4 per group), limiting the generalizability of the findings to elite professional basketball populations. Second, considerable variation existed across studies in flywheel inertia (approximately 0.010–0.075 kg·m^2^), intervention duration (4–8 weeks), training frequency (1–2 sessions per week), exercise selection, training volume, and comparator protocols. Given the limited number of available studies, reliable subgroup analyses or meta-regression could not be performed, preventing identification of an optimal FRT prescription for basketball players. Third, all included studies were judged to have some concerns regarding risk of bias, primarily related to the randomization process and selective reporting, which may have affected the robustness of the pooled estimates. Future studies should therefore employ adequately powered, preregistered randomized controlled designs across basketball players of different sexes and competitive levels, provide more standardized reporting of training prescriptions, and incorporate performance measures with greater basketball-specific relevance to further establish the practical value of flywheel resistance training. In addition, all included studies assessed outcomes only immediately after training and did not report follow-up data or adverse events, limiting conclusions regarding the durability and safety of FRT. Fourth, the eligibility criteria were restricted to peer-reviewed full-text publications and excluded gray literature, such as conference abstracts and theses. This restriction may have increased susceptibility to publication bias by limiting the inclusion of unpublished or non-peer-reviewed findings. Future studies should therefore employ adequately powered, preregistered randomized controlled designs across basketball players of different sexes and competitive levels, provide more standardized reporting of training prescriptions, and incorporate performance measures with greater basketball-specific relevance to further establish the practical value of flywheel resistance training.

## Conclusions

This study focused specifically on basketball players and systematically examined the effects of flywheel resistance training, compared with traditional resistance training, on jump, linear sprint, and COD performance. The available evidence suggests a potential additional benefit of flywheel resistance training for jump and COD performance, whereas evidence for an additional benefit on linear sprint performance remains uncertain. The differential responses across outcomes suggest that the transfer of flywheel training adaptations may be task-specific, with its benefits more likely to emerge in performance tasks involving jumping, braking, directional changes, and reacceleration in basketball, rather than as a universal replacement for traditional resistance training. Across the included studies, FRT was typically implemented for 4–8 weeks, 1–2 times per week, and predominantly involved lower-body multi-joint exercises such as squats or half squats. Accordingly, coaches and strength and conditioning practitioners may consider flywheel resistance training as a targeted complement to traditional resistance training for developing jump and COD performance in basketball players.
