# Supplementary material for: Effects of flywheel resistance training on jump, sprint, and change-of-direction performance in basketball players: a systematic review and meta-analysis
Source: Front Physiol. 2026 Oct 6;17:1989504. doi: 10.3389/fphys.2026.1989504 (PMC13681171; doi:10.3389/fphys.2026.1989504)
Supplement: Supplementary file 1 [file DataSheet1.docx]

Supplementary Table 1. Comparison of three-level and two-level random-effects models for each performance outcome

| **Model** | **df** | **AIC** | **BIC** | **AICc** | **logLik** | **LRT** | **p** | **QE** |
| --- | --- | --- | --- | --- | --- | --- | --- | --- |
| Three-level model (Jump) | 3 | 16.799 | 16.637 | 24.799 | −5.400 | — | — | 7.483 |
| Two-level model (Jump) | 2 | 14.799 | 14.691 | 17.799 | −5.400 | 0.000 | 1.000 | 7.483 |
| Three-level model (Sprint) | 3 | 23.806 | 24.045 | 29.806 | −8.903 | — | — | 16.243 |
| Two-level model (Sprint) | 2 | 22.884 | 23.043 | 25.284 | −9.442 | 1.078 | 0.299 | 16.243 |
| Three-level model (COD) | 3 | 15.445 | 14.273 | 39.445 | −4.723 | — | — | 7.212 |
| Two-level model (COD) | 2 | **13.445** | **12.664** | **19.445** | −4.723 | 0.000 | 1.000 | 7.212 |

Abbreviations: AIC, Akaike information criterion; BIC, Bayesian information criterion; AICc, corrected Akaike information criterion; logLik, log-likelihood; LRT, likelihood ratio test; QE, Cochran’s Q statistic for residual heterogeneity. Three-level and two-level random-effects models were compared using likelihood ratio tests. The three-level model was retained only when it provided a significantly better fit than the corresponding two-level model. Otherwise, the more parsimonious two-level random-effects model was selected for the primary meta-analysis.

Supplementary Table 2. Sensitivity analyses using alternative pre–post correlation coefficients for jump, sprint, and change-of-direction performance

| Outcome | r | Hedges’ g | 95% CI | p | I² |
| --- | --- | --- | --- | --- | --- |
| Jump | 0.30 | 0.56 | [0.18, 0.95] | 0.011 | 0.0% |
|  | 0.50 | 0.65 | [0.23, 1.07] | 0.008 | 0.0% |
|  | 0.70 | 0.81 | [0.31, 1.31] | 0.006 | 26.2% |
| Sprint | 0.30 | −0.49 | [−1.04, 0.05] | 0.068 | 0.0% |
|  | 0.50 | −0.57 | [−1.20, 0.05] | 0.065 | 16.0% |
|  | 0.70 | −0.71 | [−1.48, 0.07] | 0.066 | 45.3% |
| COD | 0.30 | −0.49 | [−0.92, −0.07] | 0.032 | 0.0% |
|  | 0.50 | −0.57 | [−1.07, −0.08] | 0.033 | 0.0% |
|  | 0.70 | −0.71 | [−1.34, −0.09] | 0.034 | 16.3% |

Note: r, assumed pre–post correlation coefficient used to estimate the standard deviation of change scores when not reported directly; CI, confidence interval; I², percentage of total variability attributable to between-study heterogeneity; COD, change of direction. The primary analysis assumed r = 0.50, with r = 0.30 and r = 0.70 examined in sensitivity analyses.

Supplementary Figure 1. Forest plot of the effects of flywheel resistance training on jump performance

Note: Squares represent individual effect-size estimates, with square size proportional to the relative weight of each effect size; horizontal lines indicate 95% confidence intervals (CIs). The diamond represents the pooled random-effects estimate. Positive Hedges’ g values favor flywheel resistance training (FRT). CMJ, countermovement jump; SJ, squat jump; TST, traditional strength training; TET, tempo eccentric training; TRST, traditional resistance squat training; TBT, traditional barbell training.

Supplementary Figure 2. Forest plot of the effects of flywheel resistance training on sprint performance

Note: Squares represent individual effect-size estimates, with square size proportional to the relative weight of each effect size; horizontal lines indicate 95% confidence intervals (CIs). The diamond represents the pooled random-effects estimate. Because lower sprint times indicate better performance, negative Hedges’ g values favor flywheel resistance training (FRT). TST, traditional strength training; TET, tempo eccentric training; BST, barbell squat training; TBT, traditional barbell training.

Supplementary Figure 3. Forest plot of the effects of flywheel resistance training on change-of-direction performance

Note: Squares represent individual effect-size estimates, with square size proportional to the relative weight of each effect size; horizontal lines indicate 95% confidence intervals (CIs). The diamond represents the pooled random-effects estimate. Because lower completion times indicate better performance, negative Hedges’ g values favor flywheel resistance training (FRT). COD, change of direction; TST, traditional strength training; TET, tempo eccentric training; BST, barbell squat training; TBT, traditional barbell training.
